# Surface Degradation of DGEBA Epoxy Resins Cured with Structurally Different Amine Hardeners: Effects of UV Radiation

**DOI:** 10.3390/polym16010067

**Published:** 2023-12-25

**Authors:** Cristian-Dragos Varganici, Liliana Rosu, Dan Rosu, Irina Rosca, Maurusa-Elena Ignat, Leonard Ignat

**Affiliations:** Centre of Advanced Research in Bionanoconjugates and Biopolymers, “Petru Poni” Institute of Macromolecular Chemistry, 41A Grigore Ghica Voda Alley, 700487 Iasi, Romania; varganici.cristian@icmpp.ro (C.-D.V.); rosca.irina@icmpp.ro (I.R.); mignat@icmpp.ro (M.-E.I.); lignat@icmpp.ro (L.I.)

**Keywords:** epoxy resins, DGEBA, cured DGEBA, hardeners for epoxy, epoxy UV irradiation

## Abstract

In this study, the effects of three diamine curing agents (aromatic, cycloaliphatic, aliphatic) on the photochemical behavior of bisphenol A diglycidyl ether networks were comparatively examined. In order to monitor structural changes and study the curing agents’ action mode, the cured epoxy resins were characterized before and after photoirradiation by means of Fourier-transform infrared spectroscopy, contact angle, differential scanning calorimetry, scanning electron microscopy, and energy-dispersive X-ray analysis, mass loss, and color modification measurements. Water absorption tests were also conducted. The cured epoxy resins are to be used in different multicomponent polymer materials for outdoor protection. The presence of the cycloaliphatic hardener led to reduced water absorption, and after UV irradiation, an increase in the glass transition temperature and lowest mass loss of the corresponding cured epoxy resin compared to the ones cured with aromatic and aliphatic hardener.

## 1. Introduction

Nowadays, thermosetting polymers account for 20% of global plastic production [1]. These solid non-fusible networks are obtained under the action of external factors, like temperature or UV irradiation, from a liquid or solid mixture of raw components. One or more are monomers with a functionality of at least three. Thermosets are represented by a variety of reactive systems: urea formaldehyde, unsaturated polyesters, phenolic and epoxy resins [2].

The strong curing capacity in obtaining thermosets with a vast and diverse number of hardeners conferred by the highly reactive epoxide ring, endows epoxy resins with a series of outstanding properties (i.e., adhesivity to different substrates, physicochemical, corrosion and electrical resistance, low shrinkage during hardening, flexibility), making them superior to other resin types (phenolic, polyester, melamine) and allowing them to be used in a wide variety of applications [3,4,5,6,7,8,9]. These facts, correlated with the high yield worldwide production and relatively low manufacturing costs, drive the expanding industries to invest in the development of epoxy (biobased) multicomponent polymer materials (i.e., blends, (micro/nano)composites, (semi)-interpenetrating polymer networks (S-)IPNs, matrices for reinforced composites) [1,2,10]. There exists a vast palette of epoxies used in a wide range of applications due to their excellent features [11]. Diglycidyl ether of bisphenol A (DGEBA) is one of the most produced epoxy resins on an industrial scale and may be cured with a large variety of hardener types [12,13,14,15,16,17].

However, epoxy resins are not without their drawbacks, such as brittleness, low thermal stability, reduced mechanical properties and high flammability [18,19,20]. It was also reported that epoxy resin UV degradation only occurs within a thin surface layer without tending to go in depth [21,22,23].

Epoxy resins are widely used as matrices in advanced composites, massively applied in industries such as construction, aerospace, marine and automotive for lightweight and mechanically strong parts [24]. Other applications of epoxy resins include coatings, sealants and adhesives, because of their outstanding adhesive properties and chemical resistance [25]. In construction, epoxy resins are applied in repairing and strengthening concrete and anticorrosion and wear-protection agents for steel structures [26]. In electronics, epoxies are used as encapsulates for LEDS and other electronic parts used in high-frequency and high-voltage applications, due to their dielectric strength and low dielectric constant, adhesives and environmentally protective coatings for printed circuit boards (PCBs). A vast application field of epoxy resins is the building and construction of fiber-reinforced composites for different products (e.g., airplanes, wind turbine blades, sport equipment, electric vehicles, etc.). Due to their excellent strength and stiffness, epoxies are used in applications where weight reduction and durability are extremely important, such as in composites reinforced with aramid, glass, natural fibers and carbon [27].

Over the past three decades, a wide variety of nanoparticle fillers, of both synthetic and natural origin, have also been frequently used in polymers to improve their properties [28,29,30]. Such nanoparticle fillers include carbon and silicon nanotubes [31,32,33], nanocellulose [34], montmorillonite [35], graphene [36,37], boron nitride [33] and fumed silica [38]. Even in small amounts, nanoparticles possess great features, such as high-energy surfaces, facilitating adsorption of polymer chains and improving their mechanical properties. Despite their excellent features, nanocomposites cannot always replace traditional composites, such as reinforced plastics, with a long fiber as dispersed phase, which leads to the achievement of the highest mechanical properties. Epoxy resins possess excellent adhesion to either low or high polar surfaces, thus being good candidates for obtaining nanocomposites with diverse types of nanoparticles [39,40]. There is an increase in the number of recently reported works on the obtaining of hybrid epoxy composites, i.e., having at least two reinforcing agents, such as fibers and dispersed nanoparticles of different nature to improve their anticorrosion properties [41,42,43,44].

In light of these aspects, this study is focused on the photochemical behavior of new DGEBA resins cured with three distinct hardeners (aromatic, cycloaliphatic, aliphatic). A comparison of the influence of the curing agent on the color variations during irradiation was performed. Structural modifications during irradiation were evaluated by Fourier-transfer infrared spectroscopy (FT-IR). Thermal and morphological behaviors were monitored by differential scanning calorimetry (DSC), scanning electron microscopy (SEM) and energy-dispersive X-ray analysis (EDAX). Water absorption tests were also conducted. The novelty and innovations of the study consist in finding a new cured epoxy resin with the most suitable hardener to be further used in obtaining new multicomponent polymer materials, such as (micro/nano)composites and fiber-reinforced composites.

## 2. Experimental Section

### 2.1. Materials

The hardeners for DGEBA (EP)—4,4′–diaminodiphenylsulfone (DDS) (purity ≥ 97%), 1,3–bis(aminomethyl)cyclohexane (CYDM) (98%), and octamethylenediamine (8CH_2_DA) (98%)—were purchased from Sigma-Aldrich (Darmstadt, Germany) and were used as received. The EP was a product from Sigma-Aldrich (St. Louis, MI, USA) with an epoxy equivalent weight of 0.53 equiv. per 100 g (M_n_~377 g mol^−1^), a viscosity of 15 Pa·s and density of 1160 kg m^−3^ at 25 °C.

### 2.2. Obtaining the Cured Epoxy Resins

**Scheme 1:**
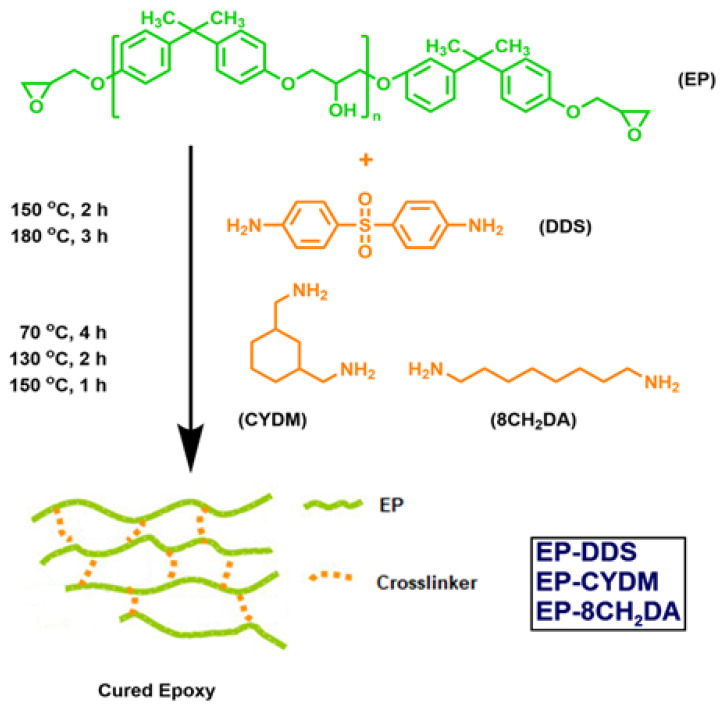
Schematic of the obtaining of the cure epoxy resins.

The epoxy resins were obtained through a facile solvent-free thermal procedure, which consists in the mixing of EP with corresponding amounts of DDS, CYDM and 8CH_2_DA (molar ratio epoxy:NH_2_ = 2:1). The homogeneous formulations were afterwards poured into polytetrafluoroethylene (PTFE)-coated plate shapes with sample dimensions of 30 mm × 30 mm × 2 mm. The hardeners’ structures dictated the thermal curing protocol. For the formulation with DDS, the protocol was 150 °C for 2 h and 180 °C for 3 h, while for the other two, it was 70 °C for 4 h, 130 °C for 2 h and 150 °C for 1 h, followed by cooling of the reaction mass to 80 °C (Scheme 1). Then, the mixtures were slowly cooled at room temperature to prevent cracking. For each sample, 2 g (0.0053 moles) DGEBA with 0.652 g (0.0025 moles) DDS, 0.368 g CYDM (0.0026 moles) and 0.378 g 8CH_2_DA (0.0025 moles) were used.

### 2.3. Measurements

#### 2.3.1. Irradiation

The photochemical behavior of the cured epoxy resins was monitored up to 500 h of UV irradiation using a UVP-B-100AP high-pressure mercury lamp (100 W) with an optical filter of maximum transparency at 365 nm (Analytik Jena, Jena, Germany) and equipped with a fan. A PMA 2100 radiometer (Solar Light Company, Glenside, PA, USA) equipped with a PMA 2110 detector (320–400 nm UVA) was used to measure the irradiance value, which was 112 W m^−2^. The radiant exposure measured for 1 h of irradiation was 162.3 kJ m^−2^, approximating 307 days of natural light into 500 h of accelerated laboratory UV exposure. This value represents the mean radiant exposure value typical for 3 months in the city of Iasi, Romania (47.19° N, 27.56° E). The UV exposure was measured in air at 25 ± 5 °C and a relative humidity of 45%. The cured resins were extracted from the irradiation chamber and analyzed every 100 h up to 500 h.

#### 2.3.2. Surface Color Change Studies

Color parameters were measured with a Lovibond LC 100 Ltd. colorimeter (Amesbury, UK). The measurements were performed in accordance with the CIE_L*a*b*_ and CIE_L*C*h*_ systems. The first color system or the Cartesian system, CIE_L*a*b*_, consists of three perpendicular axes, where parameter L^*^ is the lightness or brightness factor, which is white for value 100, black for value 0 and gray for intermediate values. Parameter a^*^ ranges between −a^*^ (shades of green) and +a^*^ (shades of red). Parameter b^*^ ranges between −b^*^ (shades of blue) and +b^*^ (shades of yellow). In the intersection zone of the three axes, the materials appear uncolored, i.e., different shades of gray [45]. The second color system or the polar system, CIE_L*C*h_^*^, uses, along with the CIE_L*a*b*_ system parameters, coordinates that quantify the angle at which the colors are observed: the values C^*^ (chroma) and h^*^ (hue), describing the chroma/saturation and the hue, which is angular, respectively. The chromatic parameters from the two systems may be correlated through the following relations:

C^*^ = (a^*2^ + b^*2^)^1/2^ (1)

h^*^ = arctan (b^*^/a^*^) (2)

Due to the variation in the color parameters L^*^a^*^b^*^ and L^*^C^*^h^*^ during UV exposure, the global color changes (ΔE_L*a*b*_ and ΔE_L*C*h*_) were estimated with the equations:

ΔE_L*a*b*_ = (ΔL^*2^ + Δa^*2^ + Δb^*2^)^1/2^ (3)

ΔE_L*C*h*_ = (ΔL^*2^ + ΔC^*2^ + Δh^*2^)^1/2^ (4)

where ΔL^*^, Δa^*^, Δb^*^, ΔC^*^ and Δh^*^ represent the differences in values L^*^, a^*^, b^*^, C^*^ and h^*^ that characterize the irradiated and non-irradiated samples. Each color value represented the arithmetic mean of five successive measurements taken from different points of the surface. Each color measurement was the mean of five individual measurements on different points of the surface. The mass loss (M) (%) after UV irradiation was calculated with the equation:

M (%) = [(M_i_ − M_d_)/M_i_] · 100 (5)

where M_i_ is the mass of initial sample and M_d_ is the mass of the sample after UV irradiation.

#### 2.3.3. Contact Angle Measurements

Static contact angle measurements of the cured resins, before and after 500 h UV irradiation, were undertaken on a CAM-200 instrument (KSV Instruments, Helsinki, Finland) with sessile drop profile analysis. The measurements were conducted at room temperature by placing a 1 μL drop of liquid on the cured resin surface. Each contact angle value was a mean of ten measurements on different surface sites.

#### 2.3.4. Structural Modifications by FT-IR during Photoirradiation

The cured resins were analyzed on a MIRacle^TM^ crystal plate for attenuated total reflectance (ATR) from diamond. The spectra were recorded between 4000 and 600 cm^−1^ with a resolution of 4 cm^−1^ and 64 scans.

#### 2.3.5. Morphological Study

The morphological study of the cured resins before and after UV irradiation was conducted on the scanning electron microscope SEM Quanta 200 (FEI Company, Hillsboro, OR, USA), operating under high vacuum mode at 30 kV with secondary and backscattering electrons.

#### 2.3.6. Differential Scanning Calorimetry

Differential scanning calorimetry (DSC) measurements were recorded on a 200 F3 Maia device (Netzsch Gerätebau GmbH, Selb, Germany): 10 mg of each sample was weighed and placed into aluminum crucibles, sealed shut with pierced lids in nitrogen as purge gas (flow 50 mL min^−1^) at a heating rate of 10 °C min^−1^.

#### 2.3.7. Water Absorption Behavior

One of the major limitations of cured epoxy resins is their tendency to absorb high amounts of water, which may affect physical properties [46]. In this sense, water absorption tests were conducted to determine the absorption parameter (Q) of the cured resins. The samples were pre-dried until constant mass at 105 °C in an oven (Memmert INB200, Borken, Germany) before performing the water absorption tests. The water was put into Erlenmeyer flasks equipped with plunger stoppers, over which the cured resins with a weight of up to 0.1 g were added at 25 °C for 240 h (10 days). The water absorption data are given in Table S1 in the Supporting Information (ESI) document. The mass of the swollen samples was measured until it was stabilized. The absorption degree was calculated with the following equation [47]:

Q (%) = [(M_w_ − M_i_)/M_i_] · 100 (6)

where Q is the absorption coefficient and M_w_ and M_i_ are the masses of the swollen and initial sample, respectively.

After 10 days in water, the EP–CYDM cured resin exhibited the lowest water absorption, with Q increasing in the order of EP–CYDM (0.16%) < EP–DDS (1.7%) < EP–8CH_2_DA (2%). The results showed no significant water uptake after the immersion period.

## 3. Results and Discussion

### 3.1. Color Modifications during UV Irradiation

**Figure 1:**
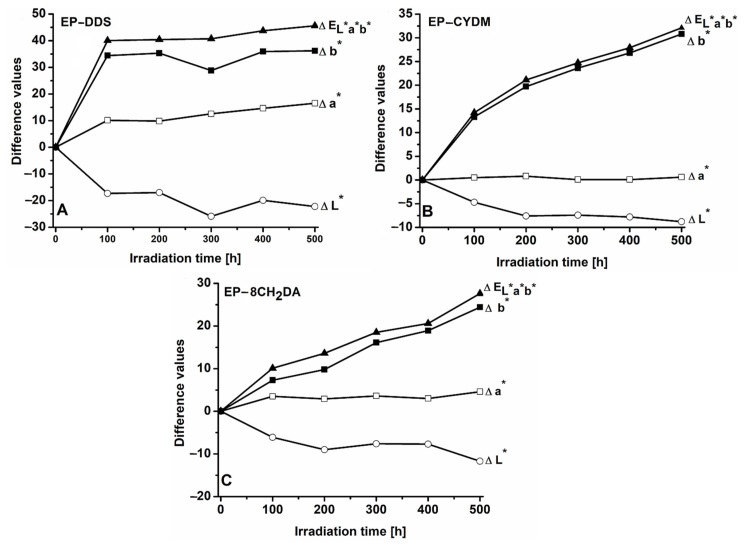
Variation in the ΔE_L*a*b*_ and the differences of color parameters with irradiation time for the cured resins in the CIE_L*a*b*_ system (**A**): EP–DDS; (**B**): EP–CYDM; (**C**) EP–8CH_2_DA.

**Figure 2:**
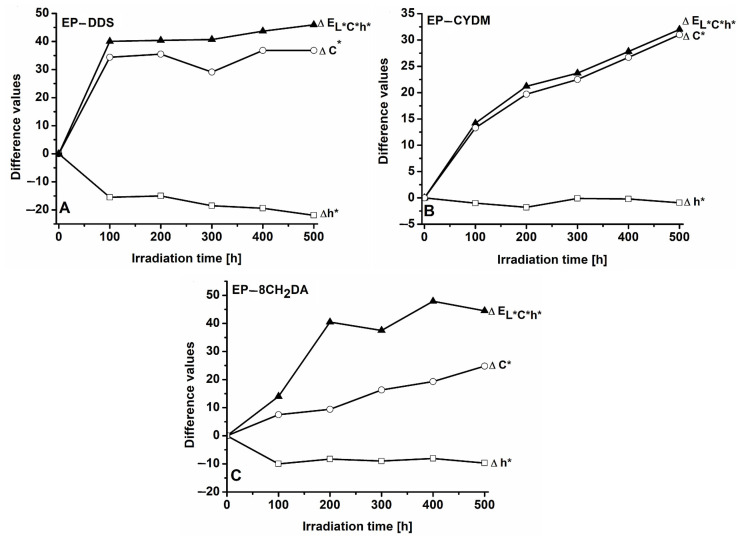
Variation in the ΔE_L*C*h_ and the differences of color parameters with irradiation time for the cured resins in the CIE_L*C*h*_ system (**A**): EP–DDS; (**B**): EP–CYDM; (**C**) EP–8CH_2_DA.

Figure 1 and Figure 2 show the variation in color parameters with irradiation time for the cured resins in both color systems: CIE_L*a*b*_ and CIE_L*C*h*_, respectively.

#### 3.1.1. Color Modifications of the EP–DDS Cured Resin

The influence of UV exposure time on the color parameters specific to the EP–DDS cured resin is presented in Table S2 (ESI). The color parameters change as a result of irradiation, and the samples becoming darker and more intensely colored with increasing UV exposure time. A facile method of monitoring the various color parameters on the surface of the cured resins under the influence of UV irradiation is achieved through observing the variations that arise in the differential parameters ΔL^*^, Δa^*^, Δb^*^, ΔC^*^ and Δh^*^ (Figure 1 and Figure 2).

The UV irradiation of the EP–DDS sample induces changes in all color parameters proportionally to the exposure time. One finding is the gradual decrease in the ΔL^*^ value. The negative values support the observation that the samples darken during exposure, the lightness factor L^*^ being lower in the case of the irradiated samples. The effect is already significant after the first 100 h of irradiation, the color difference representing 77.9% of the ΔL^*^ value obtained after 500 h of exposure. The UV exposure also affects the value of the a^*^ coefficient, which increases from negative values (−4.2) to 11.9 during the 500 h of exposure (Table S2). It can also be noted that Δa^*^ has positive values over the entire range of exposure times, sustaining the reddening tendency of the material. In the first hundred hours, the increase in the Δa^*^ value represents 61.3% compared to the value of the change in this parameter found after 500 h. Another effect of the action of UV radiation is the yellowing of the material. The positive variations in the difference Δb^*^ support the observation of the yellowing process. In this case, the maximum change occurs in the first 100 h. This represented more than 95% of the evolution of the Δb^*^ value. The variation in all color parameters with irradiation time results in the increase in the total color difference ΔE_L*a*b*_, which in the 500 h of irradiation reaches the value of 45.6 units (Table S3), of which 87.8% represents the global changes recorded in the first 100 h. Figure 2 shows the changes in the differences ΔC^*^, Δh^*^, ΔE_L*C*h*_.

Once the exposure time increases, the color saturation also increases, samples becoming more intensely colored as an effect of the UV action. On the other hand, the gradual decrease in the h^*^ value from 99.9 to 77.8 (Table S2, ESI) (Δh^*^ = −21.1) during the 500 h of photochemical treatment is indicative of color modification also underlying the presence of red chromophores. The color differences for the cured resin EP–DDS calculated with the two color systems increase rapidly in the first 100 h of irradiation, the values being comparable for ΔE_L*a*b*_ and ΔE_L*C*h*_ (Table S3, ESI). According to the literature, color changes may be classified using the ΔE values into negligible (0 < ΔE < 0.5), slightly perceivable (0.5 < ΔE < 1.5), noticeable (1.5 < ΔE < 3), appreciable (3 < ΔE < 6), and very appreciable (6 < ΔE < 12) [48,49]. The most important changes occur in the first 100 h of exposure, when ΔE values exceed 87% of those recorded after 500 h of UV exposure. It was also found that the ΔE > 40 values calculated at different irradiation times far exceed the limit of very appreciable changes (ΔE = 12), thus indicating that UV radiation strongly affects the color of the EP–DDS sample.

#### 3.1.2. Color Modifications of the EP–CYDM Cured Resin

The color parameters of the EP–CYDM cured resin undergo changes under the action of UV radiation (Table S4, ESI) and also preserve the darkening tendency of the irradiated sample surface by the gradual lowering of the L* parameter values. Likewise, there is a clear yellowing tendency of the material surface with irradiation time, signaled by the significant increase in b^*^ parameter values. The evolution of color differences with irradiation time in the CIE_L*a*b*_ system is shown in Figure 1.

As in the case of EP–DDS cured resin, there is a tendency of surface darkening for EP–CYDM cured resin throughout the 500 h exposure time, the ΔL^*^ values being negative. Darkening is more accentuated in the first 200 h when ΔL^*^ represents 86.4% of the change in the L^*^ values recorded after 500 h of exposure. The Δa^*^ parameter shows only small oscillations essentially placed within the experimental error limits (Table S4). The Δb^*^ values increase continuously with exposure time, which clearly indicates the yellowing of the sample surface. Therefore, the surface of EP–CYDM cured resin visibly changes through color darkening and accumulation of yellow chromophores. The evolution of color differences in the CIE_L*C*h*_ system is shown in Figure 2B.

The color saturation of EP–CYDM cured resin increases with irradiation time, the ΔC^*^ values being positive throughout the 500 h of irradiation (Figure 2B). During the entire exposure time, Δh^*^ variation is small and does not contribute significantly to the increase in ΔE_L*C*h*_ values. The evolution of the global color differences for the EP–CYDM cured resin expressed in the ΔE_L*a*b*_ and ΔE_L*C*h*_ values is comparatively presented in Table S5 (ESI). In the case of EP–CYDM cured resin, a rapid increase in ΔE values may be noted, especially in the first 100 h of UV exposure. Thus, over 44% of the total color change was measured after the first 100 h and about 87% after 400 h of exposure. Good agreement between the ΔE values calculated with both CIEL^*^a^*^b^*^ and CIEL^*^C^*^h^*^ systems must also be noted. A ΔE value of around 32 registered after 500 h of irradiation proves the very evident color change in the EP–CYDM surface as a result of UV exposure.

#### 3.1.3. Color Modifications of the EP–8CH_2_DA Cured Resin

The influence of UV exposure time on the color parameters of the EP–8CH_2_DA cured resin is presented in Table S6 (ESI). As in the previous two cases, the UV radiation also alters the color of the EP–8CH_2_DA sample. Thus, the brightness factor L^*^ decreases continuously with the exposure time, while the chromatic parameters a^*^ and b^*^ increase, which is reflected in the gradual increase in the index C^*^. There is also a gradual decrease in the angular index h^*^ that reflects changes in hue with irradiation time, showing an influence from newly formed red chromophores as a result of photodegradation. A semiquantitative evaluation of the influence of irradiation time on color changes is reflected in the variation pattern of ΔL^*^, Δa^*^, Δb^*^, ΔC^*^, Δh^*^, ΔE_L*a*b*_ and ΔE_L*C*h*_ with the irradiation time (Figure 1 and Figure 2).

Figure 1C shows that the EP–8CH_2_DA sample darkens during UV exposure. The ΔL^*^ value is negative during the entire irradiation period. At the same time, there is a slight increase in the difference Δa^*^, which reflects the increase in the redness–greenness coefficient, confirming the formation of red chromophores. A significant increase in the difference Δb^*^ can also be noted, indicating the change in the chromatic coefficient of yellowness–blueness, in the direction of surface yellowing. All these changes generated by the UV action on the sample are reflected in the continuous increase in the value of the total color difference ΔE_L*a*b*_, especially influenced by the yellow chromophore formation.

Figure 2C demonstrates that UV radiation causes an increase in the chroma value (C^*^) on the surface of the EP–8CH_2_DA cured resin, the sample becoming brighter once the irradiation time increases. Regarding the shade variation (Δh^*^—difference in hue), it has negative values throughout the entire UV exposure time range, indicating the lowering of the angular factor h^*^. The decrease in the numerical value of the angular size h^*^ can be seen as a reflection of the influence of red chromophores on the final color of the EP–8CH_2_DA sample surface as a result of photochemical decomposition reactions. Changes in the values of the three parameters L^*^, C^*^ and h^*^ as a result of the action of UV radiation on the sample are reflected in the gradually increasing values of the total color difference ΔE_L*C*h*_, again indicating visually obvious changes. In Table S7, the evolution of the global color differences for the EP–8CH_2_DA cured resin calculated with the ΔE_L*a*b*_ and ΔE_L*C*h*_ relations is presented comparatively. The data in Table S7 reflect the agreement between the ΔE values calculated with the two color analysis systems. It can also be observed that the main global color changes were registered in the first 100 h of irradiation when ΔE values were between 36.5% and 44.8% of those registered after 500 h of exposure. This finding underlines the presence of very obvious color changes that can be identified even after the first 100 h of exposure.

#### 3.1.4. Concluding General Remarks on Color Modifications of the Cured Epoxy Resins

The total color differences can be viewed as assessment criteria regarding the photochemical stability of polymer materials. The UV radiation with λ = 365 nm produces important color changes in the three studied samples. The color changes are proportional to the UV exposure times, being visually identifiable even after 100 h of exposure. A common feature is represented by surface darkening. The values ΔL^*^ after 500 h of exposure vary in the order EP–DDS (ΔL^*^ = −22.2) < EP–8CH_2_DA (ΔL^*^ = –11.7) < EP–CYDM (ΔL^*^ = − 8.8). The surface color increases with the irradiation time, samples becoming brighter after exposure. Chromatic differences after 500 h of UV exposure vary in the order EP–DDS (ΔC^*^ = 36) > EP–CYDM (ΔC^*^ = 30.8) > EP–8CH_2_DA (ΔC^*^ = 24.8). Positive values of ΔC^*^ indicate more intense color after exposure to UV radiation. The angles of the shade fall below 90° at the end of irradiation, indicating changes due to the increase in the influence of red chromophores after exposure. The differences in shade (Δh^*^) vary in the order EP–DDS (Δh^*^ = −22) < EP–8CH_2_DA (Δh^*^ = −9.7) < EP–CYDM (Δh^*^ = −1). There were no major differences between the total color changes calculated through the two analysis systems (CIEL^*^a^*^b^*^and CIEL^*^C^*^h^*^). The total color changes for the three studied cured resins vary in the order EP–DDS (ΔE_L*a*b*_ = 45.6, ΔE_L*C*h*_ = 45.9) > EP–CYDM (ΔE_L*a*b*_ = 32.1, ΔE_L*C*h*_ = 32) > EP–8CH_2_DA (ΔE_L*a*b*_ = 27.6, ΔE_L*C*h*_ = 29.1).

### 3.2. Structural Modifications

#### FT–IR Analysis

**Figure 3:**
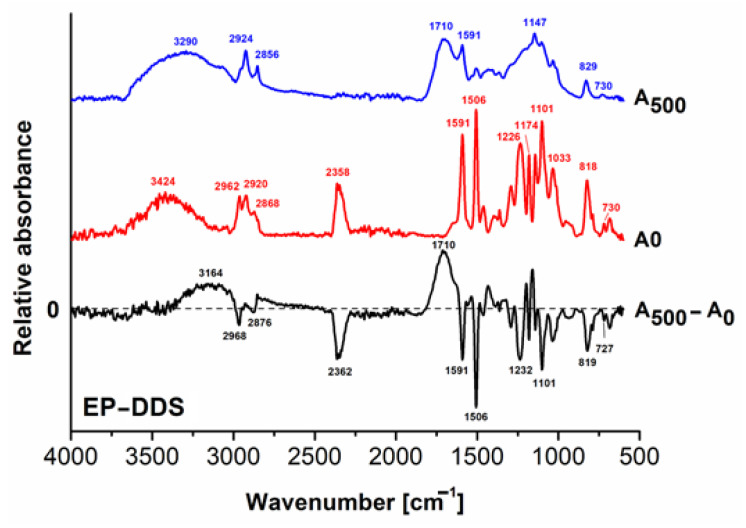
FT–IR spectra of the EP–DDS cured resin: before irradiation (A_0_); after 500 h exposure (A_500_) and the difference spectrum (A_500_ − A_0_).

**Figure 4:**
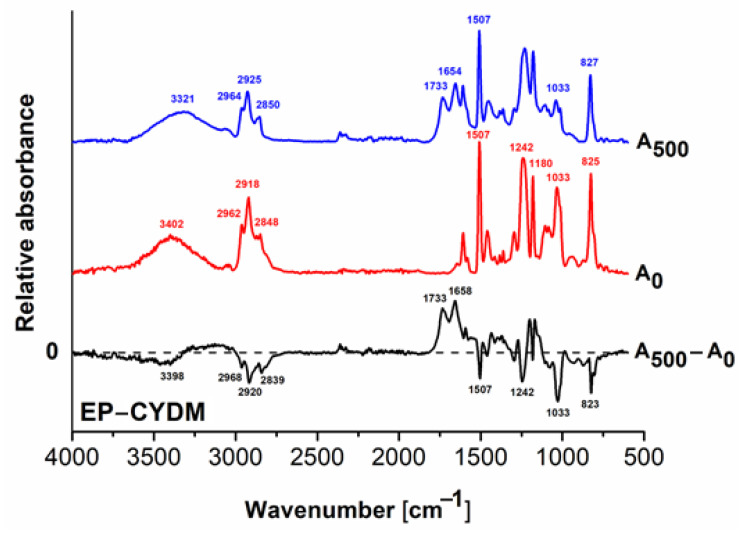
FT–IR spectra of the EP–CYDM cured resin: before irradiation (A_0_); after 500 h exposure (A_500_) and the difference spectrum (A_500_ − A_0_).

**Figure 5:**
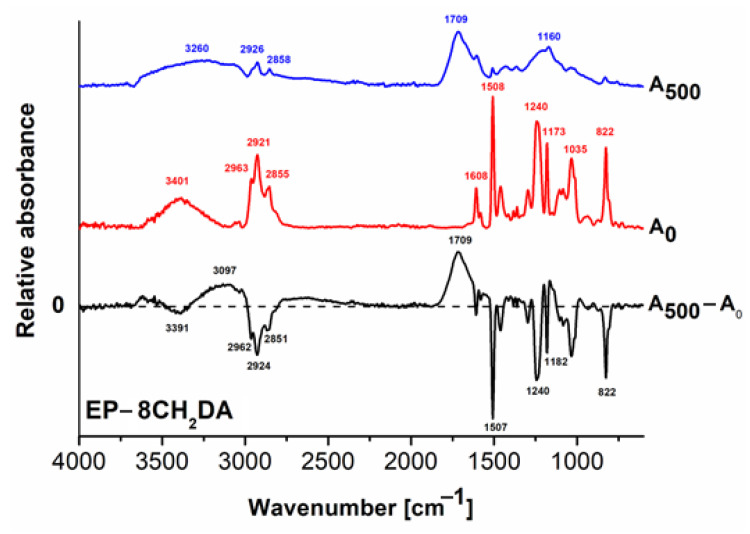
FT–IR spectra of the EP–8CH_2_DA cured resin: before irradiation (A_0_); after 500 h exposure (A_500_) and the difference spectrum (A_500_ − A_0_).

Figure 3 depicts the FT–IR spectra of the EP–DDS registered before (A_0_) and after 500 h of UV exposure (A_500_). During UV irradiation, the positive signals (upwards) in the difference spectra from Figure 3, Figure 4 and Figure 5 (A_500_ − A_0_) show the newly formed entities, and the negative signals (downwards) indicate the entities that are decomposed during UV irradiation.

The general decrease in the signals is an indication that photodegradation occurs via chain scission and mass loss [50]. According to the literature, neat DGEBA epoxy resin is characterized by the signal of the epoxy ring at 915 cm^−1^ and the signals from 1591 and 1506 cm^−1^ corresponding to skeletal C=C vibrations in aromatic moieties, while the region 1000–1300 cm^−1^ describes aryl alkyl ether entities [51]. These signals also appear in the FTIR spectra of the cured resins. The signals of 2962 cm^−1^ and 2920 cm^−1^ correspond to aliphatic C–H asymmetric stretching vibrations, and the one at 2868 cm^−1^ describes the aliphatic C–H symmetric stretching vibrations. The FT-IR spectrum of the initial EP–DDS (A_0_) shows a series of signals, both in functional group area (1500–4000 cm^−1^) and the fingerprint area (600–1500 cm^−1^). The wide signal ranging from around 3100 to 3800 cm^−1^ with the peak at 3424 cm^−1^ can be attributed to the tensile vibrations of O–H bonds generated during the reaction of the hardener (DDS) with the epoxide ring. The curing reaction between the epoxy ring and hardener is further demonstrated by the absence of epoxy characteristic absorption bands, i.e., the band at 3056 cm^−1^ from C–H stretching vibrations of methylene in the epoxy ring and the band at 915 cm^−1^. Moreover, the bands at 1174 and at 1226 cm^−1^ are specific to C–N bonds from secondary amines formed as a result of reaction with epoxy rings [52]. The signals at 2868 cm^−1^, 2920 cm^−1^ and 2962 cm^−1^ describe the valence vibrations of the CH_2_ and CH_3_ entities, while the signals from 1591 cm^−1^, 1506 cm^−1^ and 730 cm^−1^ are vibrations of the aromatic C=C bonds in the epoxy resin. The weak signal at 730 cm^−1^ is also specific to the stretching vibration of C–S bond [53] of low photochemical stability [54]. This signal almost disappears in the FT–IR spectrum recorded after 500 h irradiation (A_500_), as shown in the difference spectrum (A_500_ − A_0_) by the negative signal at 727 cm^−1^, meaning that some residual S moiety content from the cleaved C–S bonds in the DDS hardener may remain at the surface. The long peak at 2358 cm^−1^ in the initial A_0_ spectrum that completely disappears in the other two spectra is due to loss of CO_2_ traces entrapped at the surface during the obtaining of the EP–DDS cured resin [55].

By analyzing both the FT–IR spectrum of the EP–DDS recorded after 500 h irradiation (A_500_) and the difference spectrum (A_500_ − A_0_) (Figure 3), a significant decrease in signal intensity of the peak at 3424 cm^−1^ accompanied by flattening and shifting to a smaller wave number (3290 cm^−1^) can be observed. This is explained by the formation of new hydroxyl moieties and their implication in generating hydrogen bonds. The formation of photochemically unstable intermediate compounds, such as hydroperoxides, is also possible. This phenomenon takes place through photooxidation of photosensitive groups from epoxy resin and/or curing agent (–CH<, –CH_2_–, etc.) [56,57]. Hence, the epoxy chains may be cleaved in smaller fragments that are photochemically destroyed, as one may observe from the lowering in signals of CH_2_ and CH_3_ moieties in the A_500_ FT-IR spectrum (2924 cm^−1^ and 2856 cm^−1^) and the negative signals in the difference FT-IR spectrum (2968 cm^−1^ and 2876 cm^−1^) [58,59,60]. The appearance of the broad positive signal from the different FT–IR spectra in the range 1870–1610 cm^−1^ with the peak at 1710 cm^−1^ indicates a strong photooxidative effect on EP–DDS, resulting in a complex mixture of carbonyl moieties (aldehydes, ketones, anhydrides, carboxylic acids) [61]. The negative signals with peaks at 1591 cm^−1^, 1506 cm^−1^ and 727 cm^−1^ were attributed to minor changes in aromatic C=C bonds. All these observations lead to an increase in the hydrophilic character through a decrease in the water contact angle value from 61.77° to 49.38°.

Figure 4 shows the FT-IR spectra of the EP–CYDM cured resin before and after 500 h of UV exposure and their difference spectra. From Figure 4, a decreasing tendency in the signal intensities in the FT–IR difference spectrum correlated with chemical entities loss may be observed. In this sense, the negative signal from 3398 cm^−1^ indicates loss of amino groups from the curing agent. The same phenomenon may be observed for the signals in the range 2968–2839 cm^−1^, which characterize the valence vibrations of the alkyl groups, indicating scission of the main chain, which can slowly increase hydrophilicity by decreasing the water contact angle from 75.03° to 72.55°. Another observation is the decrease in the intensity of the signal located at 1507 cm^−1^ and appearance of the peak at 1654 cm^−1^ in the FT–IR spectrum of the irradiated sample (1658 cm^−1^ in the difference spectrum), describing the tensile vibrations of the C=C bonds in aromatic nuclei. The new signal at 1733 cm^−1^ after irradiation describes the formation of new carbonyl entities, hence supporting photooxidation phenomena.

Figure 5 shows the FT-IR spectra of the EP–8CH_2_DA cured resin before and after 500 h of UV exposure and the difference spectrum. The wide positive peak at 3097 cm^−1^ is assigned to new hydroxyl groups, while the wide one at 1709 cm^−1^ supports the formation of various new of carbonyl functions, such as aldehydes, ketones, organic acids and peroxides, due to photooxidation. These factors, correlated with the negative signals from C–H groups at 2962 cm^−1^, 2924 cm^−1^ and 2851 cm^−1^ originating from the 8CH_2_DA hardener, contribute to a significant decrease in the water contact angle from 78.64° to 49.12° during photoirradiation, hence strongly increasing hydrophilicity. A significant reduction in the vibration of the signal at 1182 cm^−1^ specific to the C–N bond in the cross-linked epoxy resin may also be observed. All these findings lead to the conclusion that the photodegradation of the EP–8CH_2_DA cured resin is the result of photooxidation by hydroperoxide intermediates and photochemical cleavage of the C–NH–C cross-linking points. All observations are indications of the cured resins undergoing main chain fragmentation. This is more prominent for the irradiated EP–DDS and EP–8CH_2_DA cured resins, due to the significant widening of their corresponding FT–IR spectra.

### 3.3. Morphological and Thermal Characterization

**Figure 6:**
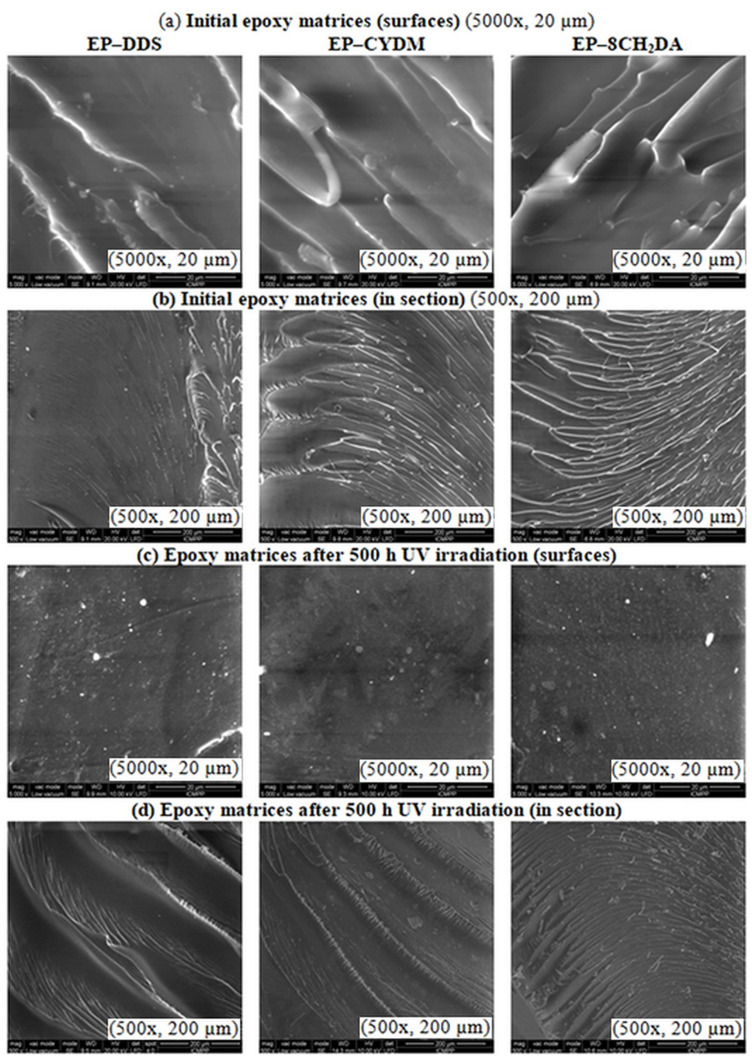
SEM micrographs of the initial cured resins: (**a**) surface and (**b**) in section; and after 500 h UV irradiation: (**c**) surface and (**d**) in section.

**Figure 7:**
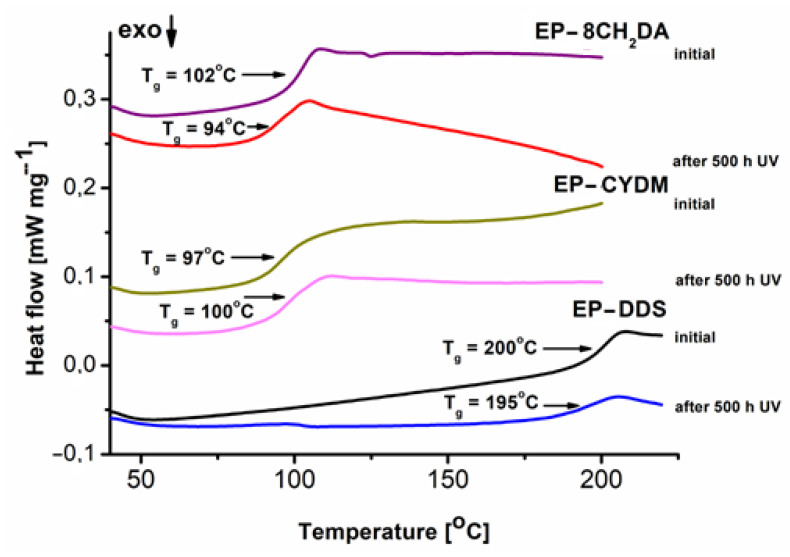
DSC curves of the cured resins before and after 500 h of UV irradiation.

SEM micrographs, both of surface and in section and the DSC curves of the initial and 500 h UV irradiated cured resins, respectively are shown in Figure 6 and Figure 7. The SEM micrographs of the initial samples indicate a smooth glassy texture with relatively parallel embossed lines, both on the surface and in section (Figure 6a,b). This, together with the absence of an exothermal profile on the DSC curves (Figure 7), is an indication of a uniform distribution of the hardener into the epoxy resin and complete reaction between the epoxide ring and amine entities from the curing agents.

All initial samples exhibit a T_g_ value dependent on the hardener type, with the highest value for the EP–DDS (200 °C) and lower values for EP–CYDM (97 °C) and EP–8CH_2_DA (94 °C) (Figure 7). After UV exposure, the T_g_ values decreased with 5 °C and 8 °C for samples EP–DDS and EP–8CH_2_DA, respectively, and slightly increased with 3 °C for EP–CYDM (Figure 7). This is an indication that the UV exposure generates damage at surface level for the cured resins, confirmed by the differences between surface SEM micrographs (Figure 6a,c) and similarity between section SEM micrographs before and after 500 h of UV irradiation (Figure 6b,d). The topography of the fractured surfaces is typical of cured epoxy resins [20]. The similarity in Figure 6b,d may indicate a possible change in the fracture mechanism, not necessarily in flexural strength.

The comparative EDAX analyses of the initial samples (Figure S1A in ESI) and the samples after 500 h UV irradiation (Figure S1B) show that the most visually affected cured resin during UV exposure is EP–DDS, with the highest C (%) loss (from 78.95% to 66.98%) and the highest O (%) increase (from 12.64% to 23.99%) due to intense surface photooxidation. The high decrease in C (%) influences the variation of other elements (e.g., S, which is not volatile at experimental temperature). Therefore, a quantitative correlation between the FT-IR and SEM-EDAX data is very difficult to obtain. The EP–8CH_2_DA cured resin exhibited lower C (%) loss (83.01% vs. 78.99%) and lower O (%) increase (12.22% vs. 15.34%) compared to EP–DDS. The lowest C (%) loss (83.03% vs. 81.78%) and lowest O (%) increase (12.12% vs. 12.92%) were registered for sample EP–CYDM.

**Figure 8:**
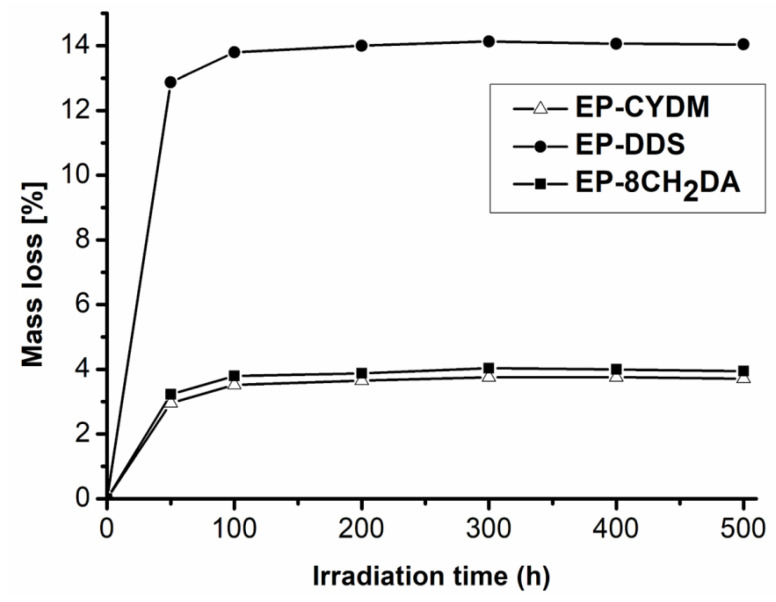
Variation of mass loss with irradiation time.

The mass loss variations in the samples measured during irradiation are summarized in Figure 8. It may be observed that the fastest mass losses were registered in the first 50 h of UV exposure, followed by a slow increase up to 100 h and reaching a stable plateau afterwards. The mass loss ranged from 2.96% for EP–CYDM (the lowest) to around 13.72% for EP–DDS (the highest) and was around 4% for EP–8CH_2_DA. After 100 h of UV exposure, there is a stabilizing tendency in the mass loss up to the end of the 500 h of UV exposure, where the values were 14.04% for EP–DDS, 3.71% for EP–CYDM and 3.95% for EP–8CH_2_DA, respectively. The lowest mass loss was registered for EP–CYDM cured resin. The large mass loss gap between the EP–DDS cured resin and the other two may be due to the destructive effect of UV on the photodegradation of the C–S bond in the DDS hardener, since it is well known that these materials have very poor resistance to UV [54]. These values also correlate well with the FTIR data, which show many negative and intense signals on the difference spectra of EP–DDS and EP–8CH_2_DA cured resins compared to that of EP–CYDM, which are much less and of lower intensity. Furthermore, the intensity and number of negative peaks in the FTIR difference spectrum of cured resin EP–DDS are much higher compared to those of the other two.

## 4. Conclusions

The comparative effect of three hardeners—4,4′–diaminodiphenylsulfone, 1,3–bis(aminomethyl)cyclohexane, and octamethylenediamine—on cured epoxy resins exposed to UV aging was studied and structural changes were monitored by surface color modification studies, FTIR, DSC, SEM–EDAX, contact angle and mass loss measurements. Water absorption tests were also undertaken. The epoxy resin cured with cycloaliphatic hardener CYDM exhibited the lowest water absorption. After UV irradiation, the same cured resin showed an increase in glass transition temperature and lowest hydrophilicity and mass loss compared to the ones cured with aromatic and aliphatic hardener. The low photochemical stability of the S–C bond in the aromatic hardener led to significant mass loss of the corresponding cured epoxy resin. All results indicate that the EP–CYDM cured resin should be further tested as a candidate to obtain new multicomponent polymer materials for different areas of applications in mainly outdoor light exposure. For example, the EP–CYDM cured resin may be used to obtain different (micro/nano)composites with metal oxides and/or fiber-reinforced composites.

## Data Availability

Data is contained within the article or supplementary material.

## Supplementary Materials

The following supporting information can be downloaded at: https://www.mdpi.com/article/10.3390/polym16010067/s1, Table S1: water absorption data; Table S2: variation of color parameters with irradiation time for EP–DDS; Table S3: color differences ΔE_L*a* b*_ and ΔE_L*C*h*_ of EP–DDS; Table S4: variation of color parameters with irradiation time for EP–CYDM; Table S5: color differences ΔE_L*a* b*_ and ΔE_L*C*h*_ of EP–CYDM; Table S6: variation of color parameters with irradiation time for EP–8CH_2_DA; Table S7: color differences ΔE_L*a* b*_ and ΔE_L*C*h*_ of EP–8CH_2_DA; Figure S1: EDAX analysis of the samples before and after 500 h UV irradiation.
