# Supplementary material for: Surface Degradation of DGEBA Epoxy Resins Cured with Structurally Different Amine Hardeners: Effects of UV Radiation
Source: Polymers (Basel). 2023 Dec 25;16(1):67. doi: 10.3390/polym16010067 (PMC10780492; doi:10.3390/polym16010067)
Supplement: Supplementary file 1 [file polymers-16-00067-s001.zip › polymers-2782094-supplementary.pdf]

SUPPORTING INFORMATION

Surface Degradation of DGEBA Epoxy Resins Cured with Structurally Different Amine Hardeners: Effects of UV Radiation

Cristian–Dragos Varganici, Liliana Rosu*, Dan Rosu*, Irina Rosca, Maurusa–Elena Ignat, Leonard Ignat

Centre of Advanced Research in Bionanoconjugates and Biopolymers, "Petru Poni" Institute of Macromolecular Chemistry, Aleea Grigore Ghica Voda, 41A, Iasi, 700487, Romania.

*authors for correspondence: Dan Rosu; E–mail: drosu@icmpp.ro

Liliana Rosu; E–mail: lrosu@icmpp.ro

1. Total number of pages: S1 – S5 (5 pages)

2. Total number of Tables: 7

3. Total number of Figures: 1

Table S1. Water absorption data

Time (h)	EP-DDS		EP-CYDM		$EP\text{-}8CH_2DA$	
	Water (g)	Q (%)	Water (g)	Q (%)	Water (g)	Q (%)
0	0.0821	-	0.1200	-	0.0399	-
5	0.0827	0.73	0.1201	0.08	0.0400	0.25
24	0.0828	0.85	0.1201	0.08	0.0401	0.75
48	0.0829	0.97	0.1201	0.08	0.0401	0.75
72	0.0831	1.21	0.1201	0.08	0.0403	1.00
144	0.0833	1.46	0.1201	0.08	0.0404	1.25
168	0.0837	1.94	0.1201	0.08	0.0404	1.25
192	0.0836	1.82	0.1201	0.08	0.0405	1.50
216	0.0836	1.82	0.1201	0.08	0.0407	2.00
240	0.0835	1.70	0.1202	0.16	0.0407	2.00

Table S2. Variation of color parameters with irradiation time for EP–DDS.

Irradiation time (h)	Color parameters									
	L*	Std.Er.	a*	Std.Er.	b*	Std.Er.	C*	Std.Er.	h*	Std.Er.
0	81.2	1.57	-4.2	0.40	23.7	0.47	24.0	0.35	99.9	0.86
100	63.5	1.94	5.5	0.20	58.1	1.63	58.4	1.54	84.4	0.55
200	64.2	0.65	5.2	0.30	59.0	1.69	59.5	0.79	84.9	0.81
300	55.4	0.22	8.0	1.25	52.5	1.98	53.1	1.57	81.4	1.24
400	61.4	1.86	10.3	1.36	59.6	3.11	60.8	1.32	80.5	1.04
500	59.0	3.65	11.9	3.20	59.9	1.62	60.8	1.42	77.8	0.36

Table S3. Color differences $\Delta E_{L^*a^*b^*}$ and $\Delta E_{L^*C^*h^*}$ of EP–DDS

**Irradiation time (h)**	$\mathbf{\Delta E_{L^*a^*b^*}}$	**(%)**	$\mathbf{\Delta E_{L^*C^*h^*}}$	**(%)**
100	40.04	87.8	40.05	87.3
200	40.4	88.6	40.4	88.01
300	40.7	89.3	40.7	88.7
400	43.7	95.8	43.7	95.2
500	45.6	100	45.9	100

Table S4. Variation of color parameters with irradiation time for EP–CYDM.

**Irradiation time (h)**	**Color parameters**									
	**L***	**Std. Er**	**a***	**Std. Er**	**b***	**Std. Er.**	**C***	**Std.Er.**	**h***	**Std.Er**
0	84.2	2.8	0.24	0.6	2.5	0.3	2.5	0.3	90.5	0.9
100	79.5	2.3	0.23	0.2	15.8	0.7	15.9	0.6	89.4	0.9
200	76.6	1.4	0.55	0.1	22.2	1.3	22.2	1.3	88.6	0.2
300	76.8	0.3	0.15	0.2	26.1	0.5	26.1	0.5	90.3	0.4
400	76.4	0.9	-0.2	0.2	29.3	1.3	29.2	1.3	90.3	1.3
500	75.4	1.8	0.4	0.2	33.3	1.2	33.3	1.2	89.5	1.0

Table S5. Color differences $\Delta E_{L^*a^*b^*}$ and $\Delta E_{L^*C^*h^*}$ of EP–CYDM

**Irradiation time (h)**	$\mathbf{\Delta E_{L^*a^*b^*}}$	**(%)**	$\mathbf{\Delta E_{L^*C^*h^*}}$	**(%)**
100	14.2	44.2	14.2	44.4
200	21.7	67.8	21.2	66.2
300	24.7	76.9	23.7	74.1
400	27.9	86.9	27.8	86.9
500	32.1	100.0	32.0	100.0

Table S6. Variation of color parameters with irradiation time for EP–8CH_2DA.

Irradiation time (h)	Color parameters									
	L*	Std.Er.	a^*	Std.Er.	b^*	Std.Er.	C^*	Std.Er.	h*	Std.Er.
0	81.2	2.3	1.5	0.4	16.4	0.8	16.3	0.8	95.4	1.2
100	75.1	0.8	1.9	0.3	23.7	0.3	23.8	0.5	85.3	0.7
200	72.2	0.5	1.4	0.3	26.2	0.8	26.2	0.8	87.1	0.8
300	73.6	1.5	2.1	0.7	32.5	1.4	32.6	1.5	86.4	1.2
400	73.2	1.7	1.5	0.4	35.3	1.4	35.6	1.7	87.3	1.6
500	69.5	1.6	3.1	1.3	41.0	1.1	41.1	1.2	85.7	1.07

Table S7. Color differences $\Delta E_{L^*a^*b^*}$ and $\Delta E_{L^*C^*h^*}$ of EP–8CH_2DA

Irradiation time (h)	$\Delta E_{L^*a^*b^*}$	(%)	$\Delta E_{L^*C^*h^*}$	(%)
100	10.1	36.5	13.9	44.8
200	13.6	49.2	15.4	52.9
300	18.2	65.9	20.1	69.1
400	20.4	73.9	22.3	76.6
500	27.6	100.0	29.1	100.0

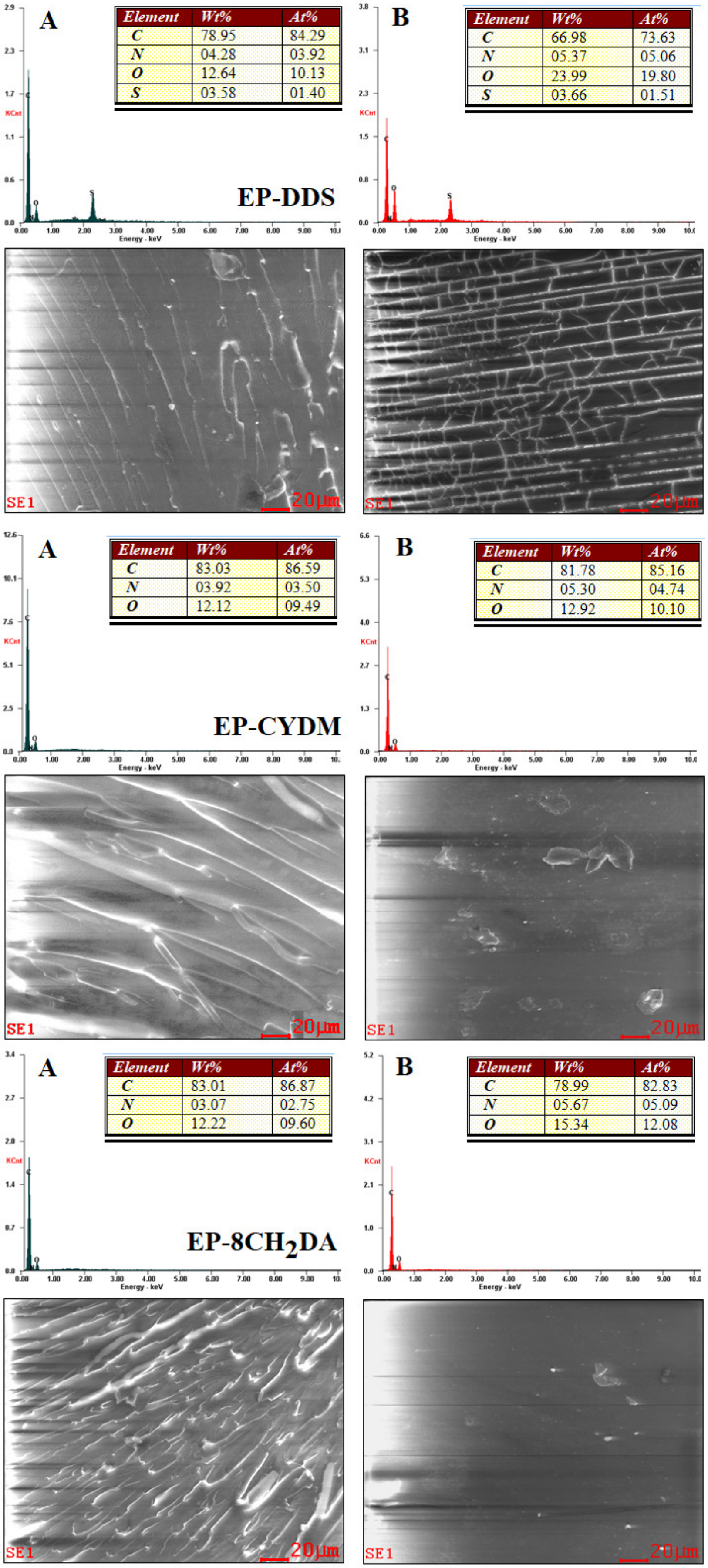

Figure S1. EDAX analysis of the samples before (A) and after 500 h UV irradiation (B)
